# *Oxynema* *mangrovii* sp. nov., a new filamentous species (Oscillatoriales, Cyanobacteria) from Atlantic forest mangrove

**DOI:** 10.3389/fmicb.2025.1709185

**Published:** 2026-01-20

**Authors:** Gladys A. Apaza-Castillo, Rafael B. Dextro, Ana P. D. Andreote, Bruno C. E. Souza, Guilherme K. Hosaka, Endrews Delbaje, Luis H. Z. Branco, Diego M. Riaño-Pachón, Marli F. Fiore

**Affiliations:** 1 Center for Nuclear Energy in Agriculture (CENA), University of São Paulo (USP), Piracicaba, São Paulo, Brazil; 2 Luiz de Queiroz College of Agriculture, University of São Paulo (USP), Piracicaba, São Paulo, Brazil; 3 Institute of Bioscience, Languages and Exact Sciences, São Paulo State University, São José do Rio Preto, São Paulo, Brazil

**Keywords:** halophiles, microcoleaceae, new species, phylogenomics, taxonomy

## Abstract

A cyanobacterial strain isolated from Brazilian mangrove soil and classified as a member of the genus *Phormidium* was afterward affiliated to the genus *Oxynema*. To define the species of this *Oxynema* strain CENA135, we sequenced its whole genome and applied a polyphasic taxonomic approach. This strain, with all the morphological features recognized for the *Oxynema* genus, had its genome assembled in 11 scaffolds with a total size of 6,235,022 bp, a G + C content of 51.6%, 4,720 protein-coding genes, and five 16S rRNA genes. Genes related to ecological resistance were annotated, demonstrating the relevance of obtaining high-quality genome assemblies from underrepresented habitats. A phylogenomic tree inferred by GTDB-Tk based on the alignment of 120 conserved proteins clustered *Oxynema* sp. CENA135 together with the strain *Oxynema aestuarii* AP17 isolated from Indian mangrove soil, and digital DNA–DNA hybridization and average nucleotide identity values between these two strains were 92.8 and 95.78%, respectively. Phylogenetic analysis based on 16S rRNA gene sequences placed the strain CENA135 in a separate and well-supported major clade (100% bootstrap) containing *Oxynema* species, and its 16S rRNA gene sequence showed identity ≤98.6% compared to the other species of the genus. Moreover, the strain CENA135 had a distinct 16S–23S ITS sequence and secondary structure polymorphisms in comparison to the other *Oxynema* species, supporting its recognition as a novel species. On the basis of evidence from this polyphasic study, strain CENA135 should be designated as representing a novel species of the genus *Oxynema*, for which the name *Oxynema mangrovii* sp. nov. is proposed under the provisions of the International Code of Nomenclature for algae, fungi, and plants.

## 1 Introduction

The genus *Oxynema* was erected with members previously classified as a special group (*Phormidium* cluster I, Komárek and Anagnostidis, 2005) within the traditional and polyphyletic genus *Phormidium* (Chatchawan et al., 2012). The description of *Oxynema* was based on detailed studies using a polyphasic approach, which mainly included differences in the trichome morphology, terminal cell shape, preference for salinity, and 16S rRNA gene sequence phylogeny (Chatchawan et al., 2012). Members of *Oxynema* are classified in the order Oscillatoriales and were previously placed under Microcoleaceae (Komárek et al., 2014), but updated to the family Oscillatoriaceae according to the system proposed by Strunecký et al. (2023).

According to AlgaeBase (Guiry et al., 2014), checked in September 2025, four species are so far described in the genus: *Oxynema thaianum* Chatchawan et al., *O. acuminatum* Chatchawan et al., *O. lloydianum* Chatchawan et al. (2012), and *O. aestuarii* Chakraborty et al. (2018). The reference strain for the genus is *O. thaianum* CCALA 960, isolated from saltworks in Petchaburi Province, Thailand (Chatchawan et al., 2012). *O. acuminatum* and *O. lloydianum* were described based on the reevaluation of type material *Oscillatoria acuminata* (synonymous with *Phormidium acuminatum*, Anagnostidis and Komárek, 1988) and *Oscillatoria lloydiana* (synonymous with *Phormidium lloydianum*, Anagnostidis and Komárek, 1988) exsiccates available in the Gomont herbarium (Herb. Crypt. Museum Paris, PC) and based on ecological and morphological similarities (Chatchawan et al., 2012). *Oxynema acuminatum* was reported from Euganean thermal springs, Italy, and *O. lloydianum* was originally described in 1899 from samples of saline and brackish localities in Bretagne, France (Anagnostidis and Komárek, 1988). The species *O. aestuarii* AP17 and AP24 were isolated from soil samples of the Sundarbans mangrove of Lothian Island and Sagar Island, respectively, in India (Chakraborty et al., 2018). Occurrences of strains of the genus *Oxynema* have also been reported in coastal areas in India (Bhuvaneshwari et al., 2016), in saline soil in Iran (Hokmollahi et al., 2017), in a hypersaline lagoon in Brazil (Ramos et al., 2017), and in an estuary–marine transition zone in Portugal (Ramos et al., 2018). So far, strains of the genus *Oxynema* have been recorded from halophilic habitats, less frequently from thermal springs and soil biotopes with higher salt contents (Chatchawan et al., 2012).

Currently, only the genome of *O. aestuarii* AP17, isolated from an Indian mangrove intertidal soil surface, has been sequenced (Basu et al., 2020). The availability of new genomes can further improve the evolutionary standing of poorly represented genera and habitats (Dextro et al., 2021) and their placement within the Genome Taxonomy Database (GTDB). GTDB is an initiative to standardize microbial taxonomy based on genome phylogeny, inferred from the concatenation of 120 conserved single-copy proteins (Parks et al., 2018).

In a 2006 survey of cyanobacterial diversity in a pristine mangrove lying within Brazilian Atlantic Forest, a strain was identified as pertaining to the genus *Phormidium*, but to an undetermined species, and then named *Phormidium* sp. CENA135. 16S rRNA phylogenetic analysis placed CENA135 loosely related to the sequence of *P. pseudopristleyi* ANT. ACEV5.3 (Silva-Stenico et al., 2012). Subsequently, the CENA135 strain gave support to the establishment of the genus *Oxynema* based on its halophilic growth, similar morphology, and 16S rRNA gene sequence (Chatchawan et al., 2012). Therefore, this strain was further renamed *Oxynema* sp. CENA135 (Silva et al., 2014). Studies have shown the important biotechnological potential of *Oxynema* sp. CENA135 as a degrader of different textile dyes (Silva-Stenico et al., 2012); producer of heptadecane, 2-hexadecene, 3, 7, 11, 15-tetramethyl-, [R-[R*, R* − (E)]], and 1-octadecyne (Armstrong et al., 2019); anticancer activity against murine colon cancer CT-26, lung carcinoma 3LL, acute myeloid leukemia MOLM-13; as well as activity against *Salmonella typhimurium* (Silva-Stenico et al., 2013; Shishido et al., 2020). Thus, the purpose of this study is to define the specific identity of *Oxynema* sp. CENA135 by a polyphasic approach, including phylogenomics, 16S rRNA gene phylogeny, 16S–23S ITS primary sequence and secondary structures, along with morphological and ecological features.

## 2 Materials and methods

### 2.1 Cyanobacterium strain, morphology, and ecology

The CENA135 strain was isolated in previous studies from a mangrove soil sample collected on April 20, 2006, at Cardoso Island, municipality of Cananéia, São Paulo state, Brazil (25°05′02”S, 47°57′42”W, Supplementary Figure S1) (Silva-Stenico et al., 2012; Silva et al., 2014). Detailed isolation information can be found in Silva et al. (2014). This strain is kept at the Center for Nuclear Energy in Agriculture/University of São Paulo in a 125-mL Erlenmeyer flask containing 50 mL of ASM-1 liquid medium (Gorham et al., 1964), in a growth room with white fluorescent irradiation (40–50 μmol photons·m^−2^·s^−1^) for a 14/10-h light/dark cycle, at 23 ± 1 °C, pH 6.8–7.2, and humidity of 60 ± 5%. Subsamples of the unicyanobacterial culture were preserved in 4% formaldehyde (*v/v*) and deposited in the “Maria Eneyda P. Kauffman Fidalgo” Herbarium (SP) of the Institute of Botany, São Paulo state, Brazil (CENA135 – voucher SP 428477).

The morphology of CENA135 was evaluated using an Olympus BX53 microscope (Olympus Optical Co., Ltd., Tokyo, Japan) equipped with a differential interference contrast device (DIC). Microphotographs and measurements were taken using a DP71 digital camera (Olympus Optical Co., Ltd., Tokyo, Japan) coupled to the optical system and the cellSens image analysis system (Olympus). Taxonomic features, such as filament structure, absence of sheath, cell shape, dimensions, and apical cell shape, were analyzed in at least 30 trichomes.

### 2.2 Cell washing and *de novo* genome assembly

Prior to total genomic DNA extraction for genome sequencing, a procedure was carried out in order to reduce associated bacterial contaminants growing in the unicyanobacterial culture. Cells from 50 mL of a 60-day-old culture were subjected to a serial washing procedure (Heck et al., 2016) modified by increasing EDTA to 5 mM and ethanol to 60% in the washing solution, and by the introduction of a final step with the resulting pellet being washed with a solution of 5 mL of 0.1% Extran and 20 mL of 0.9% NaCl by vacuum filtration through an 8-μm nitrocellulose membrane. Total genomic DNA was extracted using the AllPrep DNA/RNA Mini Kit (Qiagen, Hilden, Germany), and a mate-pair library was prepared from 5 to 8 kbp inserts using the Nextera Mate Pair Library Prep Kit (Illumina), according to the manufacturer’s protocols. Sequencing was carried out on the HiSeq 2,500 platform (2 × 100 bp) using the HiSeq v4 Reagent Kit (Illumina) following the instructions provided by the manufacturer. Adapters of the mate-pair library and non-mate-pair reads were removed from datasets using NxTrim v0.4.2 (O’Connell et al., 2015). Then reads shorter than 30 bp were removed with Cutadapt v1.18 (Martin, 2011). The Phred Quality Score (PQS) was not considered as a filter because all bases presented a PQS greater than 22. Jellyfish v. 2.2.10 (Marçais and Kingsford, 2011) was employed to count and determine the k-mer distribution with lengths 21 and 33 bp. The k-mer profiles, genome estimates, and heterozygosity rates were obtained with GenomeScope v.2.0 (Ranallo-Benavidez et al., 2020).

Individual assemblies were generated with (i) SPAdes v3.13.0 (Bankevich et al., 2012), using error correction and automatic k-mer estimation size; (ii) Platanus 1.2.4 (Kajitani et al., 2014) with default parameters; and (iii) a bin obtained using SPAdes through the metagenomic pipeline metaSPAdes, complemented with MetaBAT2 v2.12.1 (Kang et al., 2019), for contig binning with the specific setting (−-specific). Contigs with less than 100 × coverage were removed from the genome assemblies. BlobTool v1.0.1 (Laetsch and Blaxter, 2017) was used to identify cyanobacterial contigs and for filtering non-cyanobacterial sequences. The resulting assemblies were merged into a single assembly with Metassembler v1.5 (Wences and Schatz, 2015) using the assembly derived from SPAdes as the principal assembly. The scaffolding and gap-closing processes were performed by Platanus, followed by polishing using Illumina reads with Pilon v1.23 (Walker et al., 2014). We used default parameters for Platanus and Pilon. Genome assembly metrics were computed with QUAST v3.2 (Gurevich et al., 2013), while the completeness and contamination assessment were estimated with CheckM v1.0.7 (Parks et al., 2015). The completeness of the genome was validated by BUSCO v3.0 (Benchmarking Universal Single-Copy Orthologs) with the database *cyanobacteria*_odb9 (Simão et al., 2015).

### 2.3 Genomic taxonomy, phylogenomic, and genome annotation

Digital DNA–DNA hybridization (dDDH) values were calculated between the *Oxynema* sp. CENA135 genome and the only currently available genome of this genus (*O. aestuarii* AP17, RefSeq assembly accession: GCF_012295525.1) using the Genome-to-Genome Distance Calculator (GGDC) version 2.0,^1^ following the recommended settings (Meier-Kolthoff et al., 2013). Furthermore, the whole-genome average nucleotide identity (ANI) value was calculated between *Oxynema* sp. CENA135 and *O. aestuarii* AP17 using OrthoANI v1.4 (Lee et al., 2016). The percentage of conserved proteins (POCP) analysis was calculated based on the description of Qin et al. (2014).

The phylogenomic tree was inferred using GTDB-Tk v0.3.2 (Chaumeil et al., 2019) based on 120 bacterial orthologous protein-coding genes. Multiple sequence alignments (MSA) from our studied genome and 230 cyanobacterial genomes available in the Genome Taxonomy Database (GTDB) were used to construct a Maximum Likelihood tree with the WAG + T model and 1,000 bootstrap replicates (Katoh et al., 2019).

After obtaining the genome assembly, the NCBI Prokaryotic Genome Annotation Pipeline (PGAP) was used to automatically annotate coding regions (CDS) within the genome (Tatusova et al., 2016). Genes related to ecological traits relevant to a halophytic tropical habitat, such as cation:anion antiporters, heat-shock response, and metalloproteins, were singled out and had their putative protein evaluated via amino acid sequence BLASTp on NCBI. Additionally, an antiSMASH v8.0.4 search was also performed (Blin et al., 2025).

### 2.4 16S rRNA phylogeny and 16S-23S ITS secondary structure

The 16S rRNA gene and the 16S–23S ITS rRNA operon of strain CENA135 were recovered from the assembled genome. It should be mentioned that the five complete copies of the 16S rRNA gene with 1,487 bp showed 100% identity with the 16S rRNA gene sequence of the same strain previously obtained by PCR amplification followed by Sanger sequencing (GenBank accession number HQ730084, Silva et al., 2014). The 16S rRNA sequence of *Oxynema* sp. CENA135 and closely related cyanobacteria were aligned to the consensus bacterial secondary structure model using SSU-Align (v0.1.1).^2^ The alignment was used to reconstruct phylogenetic trees using maximum likelihood (ML) and bayesian inference (BI). The ML tree was constructed using RAxML v8.2.12 (Stamatakis, 2014) by applying the best-fitted evolutionary model GTR + I + C and bootstrap analysis using 1,000 replicates. Bayesian inference was conducted with MrBayes v.3.2.1 (Ronquist et al., 2012), employing the GTR + IΤ + I model. Two separate runs with four chains were performed for 5 × 10^7^ Markov Chain Monte Carlo generations. For this method, the first 0.1% of trees were discarded, and the phylogenetic tree with the highest posterior probability was selected. Phylogenetic trees obtained by the ML and BI methods were visualized in ITOL 5.3 (Letunic and Bork, 2019), showing a similar topology. Hence, only the ML tree is displayed, along with the bootstrap resampling values and the Bayesian posterior probabilities. Furthermore, the 16S rRNA gene sequences of *Oxynema* strains were aligned with SSU-Align and then sliced for a consensus of 1,064 to 1,067 bp. The identity matrix was elaborated by using BioEdit v. 7.2.5 (Hall, 1999).

The 16S–23S ITS sequence of the studied strain was used for secondary structure folding in order to support recognition of *Oxynema* sp. CENA135 as a new species. The 16S–23S ITS regions were identified using LocARNA-Alignment and Folding (Will et al., 2007; Will et al., 2012; Raden et al., 2018), and the tRNA genes were identified using the tRNAscan-SE Search Server (Lowe and Chan, 2016). The secondary structures (D1-D1′, Box-B and V3) were folded using the Mfold WebServer v.3.6 with default conditions (Zuker, 2003).

## 3 Results and discussion

### 3.1 Morphological aspects

**Figure 1:**
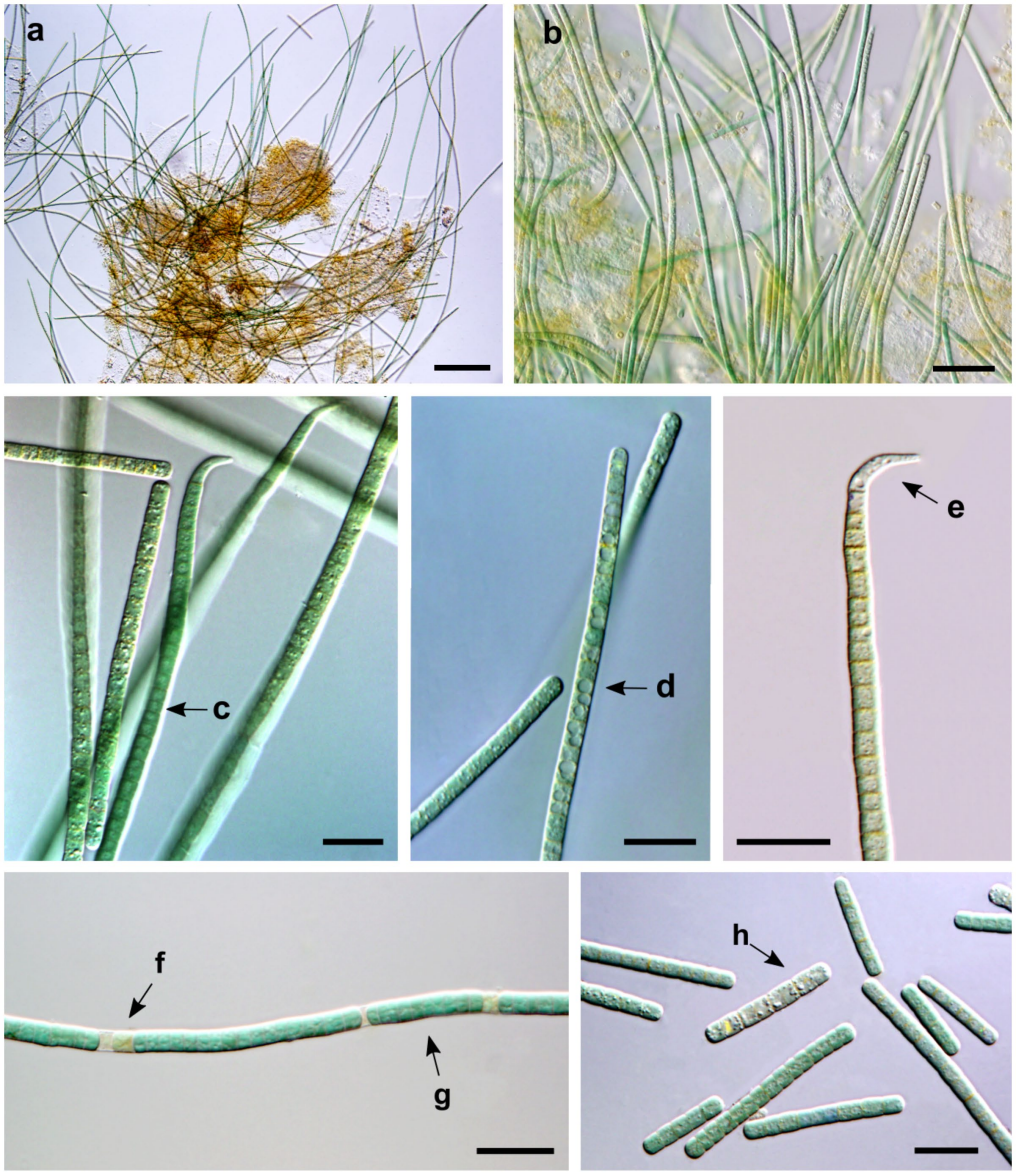
Microphotographs of *Oxynema mangrovii* sp. nov. CENA135 **(a–h)**. **(a,b)** Arrangement of long and entangled trichomes. Arrows indicate **(c)** vegetative cells shorter than wide, **(d)** central keritomic vacuole in vegetative cell, **(e)** curved apical cell, **(f)** necridial cell, **(g)** hormogonia formation, **(h)** developing short trichomes. Bars, 100 μm **(a)**, 20 μm **(b)**, 10 μm **(c–h)**.

The CENA135 strain isolated from Brazilian mangrove wet soil shares diagnostic features of *Oxynema* according to the original description of Chatchawan et al. (2012). Trichomes of the CENA135 strain grew entangled (Figures 1a,b), forming blue–green mats with a deep blue color in low-light conditions. This strain showed trichomes straight or slightly flexuous, attenuated at the ends and constricted at the cross-walls. Vegetative cells were generally cylindrical, 1.9–5.1 μm long (3.3 μm on average), 2.7–3.4 μm wide (3.1 μm on average), and had a cell length/width ratio of 0.6 to 1.1 (sometimes isodiametric) (Figure 1c). Cell dimensions of the CENA135 strain were smaller than the reference strain *O. thaianum* CCALA 960 (2.2–3 μm long and 7.5–9 μm wide) (Chatchawan et al., 2012) and slightly larger than *O. aestuarii* strains AP17 and AP24 (Chakraborty et al., 2018). A central keritomic vacuole was observed in some cases, especially in mature cells (Figure 1d). The apical cells were elongated, narrowed, usually pointed, and bent (Figure 1e). This is an autapormorphic characteristic of the *Oxynema* genus as it was diacritical in the separation of “Group I-*Phormidium*” (Komárek and Anagnostidis, 2005). Necridial cells were observed before the trichomes broke into hormogonies (Figures 1f-h). The trichomes were intensely mobile by gliding. The rapid displacement in the troubled mangrove by tides would allow the search for the best positioning in relation to the source of light.

### 3.2 Genome features and phylogenomic analysis

The k-mer profiles (k = {21, 35}), fitted to GenomeScope model, showed a single peak, suggesting a haploid genome (Supplementary Figure S3). Heterozygosity rates were close to zero, demonstrating that genetic diversity among cloned individuals was extremely low. A draft genome was obtained from the consensus of the Spades, Platanus, and MetaBAT2 (binning method) assemblies. Metrics of individual and consensus assemblies are summarized in Supplementary Table S1. The final genome, once corrected by Pilon, had 11 scaffolds, with N50 of 3,585,021 bp and a total length of 6,235,022 bp (Table 1). This genome consisted of a total of 4,897 genes, including 93 RNA genes (15 rRNAs, 74 tRNAs, and 4 non-coding RNAs) and 4,804 CDSs (4,720 protein-coding genes and 84 pseudogenes). We achieved a completeness of 99.3% in CheckM analysis, and 99% of BUSCOs were detected (826 complete BUSCOs) (Table 1).

**Table 1:** Summary statistics of *Oxynema mangrovii* CENA135 genome.

Parameters	Values
Assembly length (bp)	6,235,022
Total number of scaffolds	11
Number scaffolds ≥ 1,000 bp	9
N50 (bp)	3,585,021
L50	1
GC content %	51.6
CheckM analysis	
Percentage of completeness	99.3
Percentage of contamination	1.22
BUSCO analysis	
Total BUSCO groups searched	834
Total complete BUSCOs	826
Complete BUSCOs and single-copy BUSCOs	821
Complete and duplicated BUSCOs	5
Fragmented BUSCOs	4
Missing BUSCOs	4

The dDDH value (62.8%) provided initial support for the description of a new *Oxynema* species since it was lower than the 70% threshold for bacterial species delineation (Stackebrandt and Goebel, 1994). ANI has been widely used and accepted for species demarcation (Konstantinidis and Tiedje, 2005; Richter and Rosselló-Móra, 2009). The *Oxynema* sp. CENA135 genome showed an ANI value of 95.78% with the *O. aestuarii* AP17 genome (Supplementary Figure S4), which is below the 96% threshold for species boundary (Ciufo et al., 2018), indicating that these genomes are distinct species. Additional genomes used in the analysis further corroborate *Oxynema* sp. CENA135 taxonomical distance from related genera, such as *Laspinema* (*L. palackyanum* D2c 78.4% and *L. olomoucense* D3b 80.53%), *Phormidium* (*P. ambiguum* IAM M71 75.52% and *Phormidium* sp. LEGE 05292 78.2%), and *Oscillatoria* (*O. acuminata* PCC 6304 80.43% and *O. princeps* RMCB10 77.99%). The percentage of conserved proteins (POCP) analysis between CENA135 and AP17 was 91.11%, surpassing 50%, which is considered a boundary for grouping prokaryotes of the same genus (Qin et al., 2014), confirming that both strains belong to the *Oxynema* genus.

**Figure 2:**
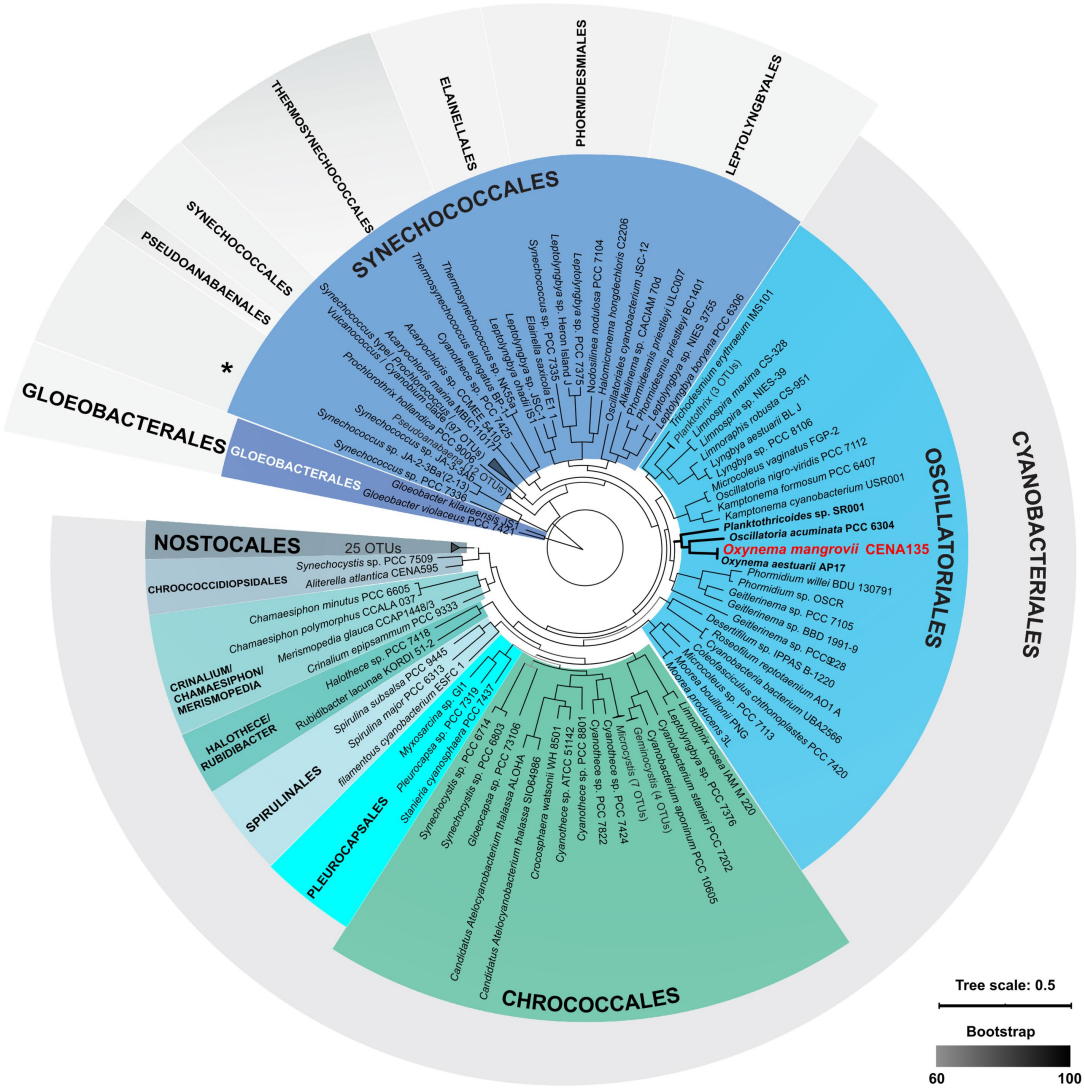
Phylogenomic tree showing the evolutionary position of *Oxynema mangrovii* CENA135 (in red). The taxonomic classification at the order level was designated according to the taxonomy used in the NCBI (first layer) and the GTDB taxonomy (Parks et al., 2019, second layer).

The phylogenomic tree depicted *Oxynema* sp. CENA135 together with *O. aestuarii* AP17 within a major clade that includes *Oscillatoria acuminata* PCC 6304 and *Planktothricoides* sp. SR001, isolated from hot springs in the United States and from a freshwater reservoir located in Singapore, respectively (Figure 2). Morphometric, ecological, and genomic comparisons between *O. aestuarii* AP17, *O. thaianum* CCALA960, and *O. mangrovii* sp. nov. CENA135 are provided in Supplementary Table S2, exemplifying some of the plasticity observed in the *Oxynema* genus.

Interestingly, the reference strain *Oscillatoria acuminata* Gomont was renamed *Oxynema acuminatum* (Chatchawan et al., 2012), but species delineation based on dDDH was unsuccessful for “*Oscillatoria” acuminata* PCC 6304 genome and *Oxynema* sp. CENA135 genome due to low values (19.6–24.3%). However, the POCP between these two strains was 65.46%, a value above the threshold (50%) for genus demarcation. This false match reveals the difficulties in using a single stationary threshold for all prokaryotes, and more discriminatory power to reflect genus boundaries may be achieved by defining different thresholds in accordance with well-established taxa (Barco et al., 2020). As observed in the phylogenetic analysis based on a larger number of 16S rRNA gene sequences discussed below and the ANI values of ~90%, the strain “*Oscillatoria” acuminata* PCC 6304 would maybe fit into the *Laspinema* clade, as previously indicated in the literature (Dvořák et al., 2024).

The phylogenomic tree displayed the classification adopted by the NCBI taxonomy database and the classification system of GTDB (Parks et al., 2019). In these systems, the strain CENA135 was positioned within the order Oscillatoriales (Komárek et al., 2014; Strunecký et al., 2023) or within the order Cyanobacteriales (Parks et al., 2019). Cyanobacteria classification is still challenging and requires continuous adaptations according to new discoveries and the introduction of new methodological approaches (Komárek, 2018; Garcia-Pichel et al., 2020).

### 3.3 16S rRNA phylogeny and 16S-23S ITS secondary structure analyses

The comparison of 16S rRNA gene sequence among *Oxynema* strains showed that *Oxynema* sp. CENA135 had 98.4% identity with *O. thaianum* CCALA960 and ≤98.6% identity with other members of the genus (Table 2). These values are below the cut-off index of 98.7% used for species delimitation of prokaryotes (Yarza et al., 2014), evidencing that CENA135 is a different species.

**Table 2:** Identity comparison of *Oxynema* species based on 16S rRNA gene sequence.

	Strain	1	2	3	4	5	6	7	8	9
1	***Oxynema mangrovii* CENA135**									
2	*Oxynema thaianum* CCALA960	98.4								
3	*Oxynema thaianum* BDU 92071	98.6	99.6							
4	*Oxynema thaianum* BDU 70493	98.6	99.5	99.9						
5	*Oxynema thaianum* BDU 10251	98.4	99.2	99.6	99.7					
6	*Oxynema thaianum* BDU 91992	98.4	99.9	99.5	99.6	99.3				
7	*Oxynema thaianum* BDU 41202	98.3	99.8	99.4	99.5	99.2	99.9			
8	*Oxynema thaianum* BDU 120161	98.4	100	99.6	99.5	99.2	99.9	99.8		
9	*Oxynema aestuarii* AP17	98.4	100	99.6	99.5	99.2	99.9	99.8	100	
10	*Oxynema*_*aestuarii* AP24	98.4	100	99.6	99.5	99.2	99.9	99.8	100	100

Sequence of the strain studied is in bold.

**Figure 3:**
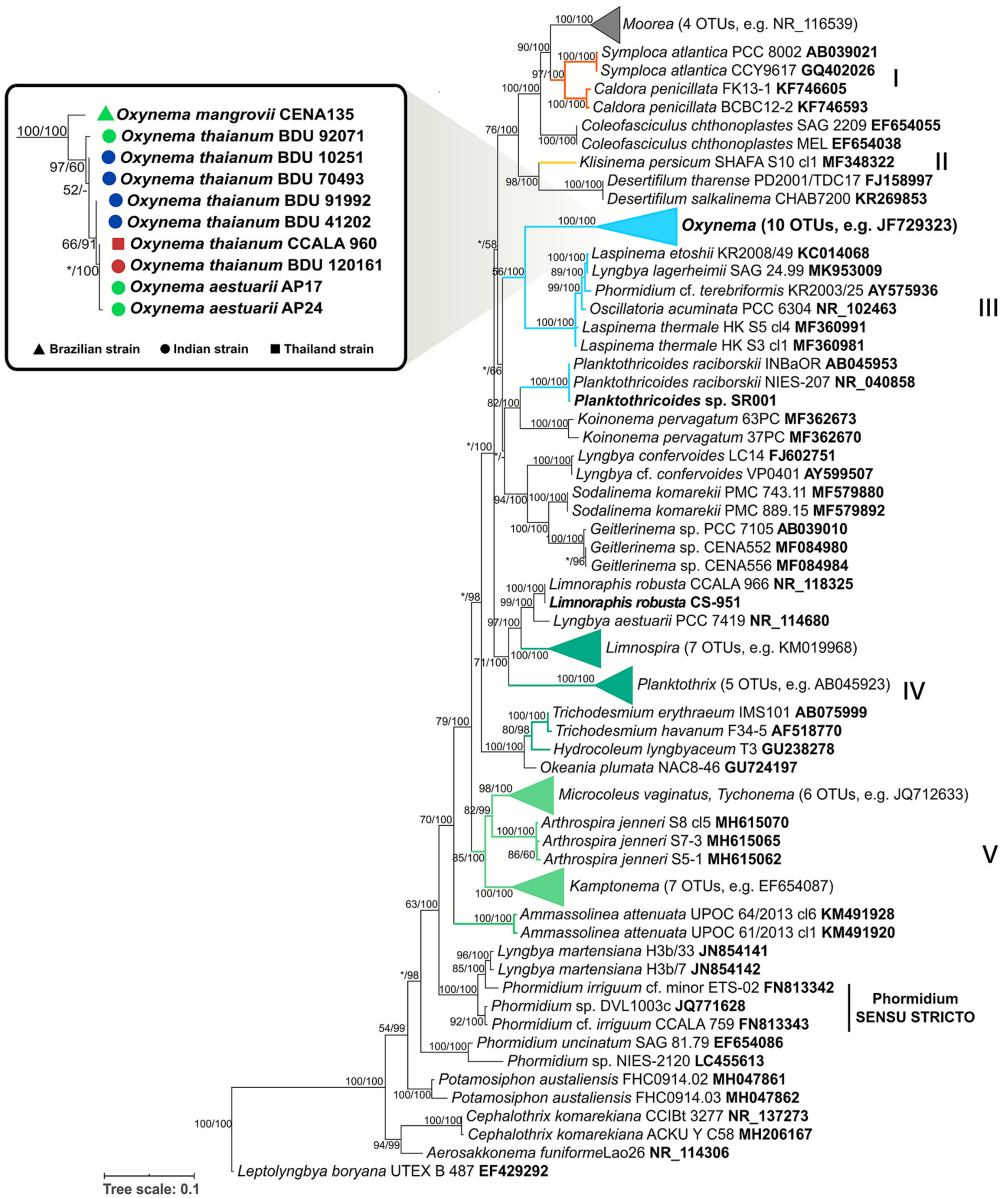
Phylogeny of the *Oxynema* strains based on the 16S rRNA gene. The maximum likelihood (ML) tree was based on partial sequences of the 16S rRNA gene, and the topology was validated by Bayesian inference (BI). Only bootstrap values greater than 50% for ML and BI are indicated in the nodes. (*) Represents lower values and (−) missing values. *Oxynema* clade symbols were colored according to the isolation habitat: mangrove or estuary (green), sea (blue), and saltworks (red).

The topology of ML and BI phylogenetic trees showed a well-supported *Oxynema* clade (100% of bootstrap) and distanced from *Phormidium sensu stricto* clade (*Phormidium* cluster VIII, Komárek and Anagnostidis, 2005) represented by strains of *P. irriguum* CCALA 759 and *P.irriguum* cf. *minor* ETS-02 (Sciuto and Moro, 2015). The *Oxynema* clade was divided into two subclades (I and II), consistent with geographical distribution (Figure 3). The subclade I included only CENA135 isolated from Brazilian mangrove, and subclade II was formed by nine Asian strains of the *Oxynema* genus, including the reference strain *O. thaianum* CCALA960 collected from saltworks in Thailand (Chatchawan et al., 2012). The two *O. aestuarii* strains (AP17 and AP24), recovered from soil biofilms in the Sundarbans mangrove in India (Chakraborty et al., 2018) and similar in morphology to the CENA135 strain, were also in subclade II. In the phylogenetic tree, the genus most closely related to *Oxynema* was *Laspinema*, recently separated from *Phormidium* (Heidari et al., 2018). Contrary to previous reports that *Oxynema* is immotile (Heidari et al., 2018), the *Oxynema* sp. CENA135 strain exhibited highly motile trichomes with conical and bent apical cells, similar to the type strain of the genus *Laspinema thermale,* HK S5, isolated from hot springs. As mentioned above, the strain “*Oscillatoria” acuminata* PCC 6304 would be better positioned into the *Laspinema* clade, and future detailed studies may provide more evidence for transferring this strain into this established genus.

**Figure 4:**
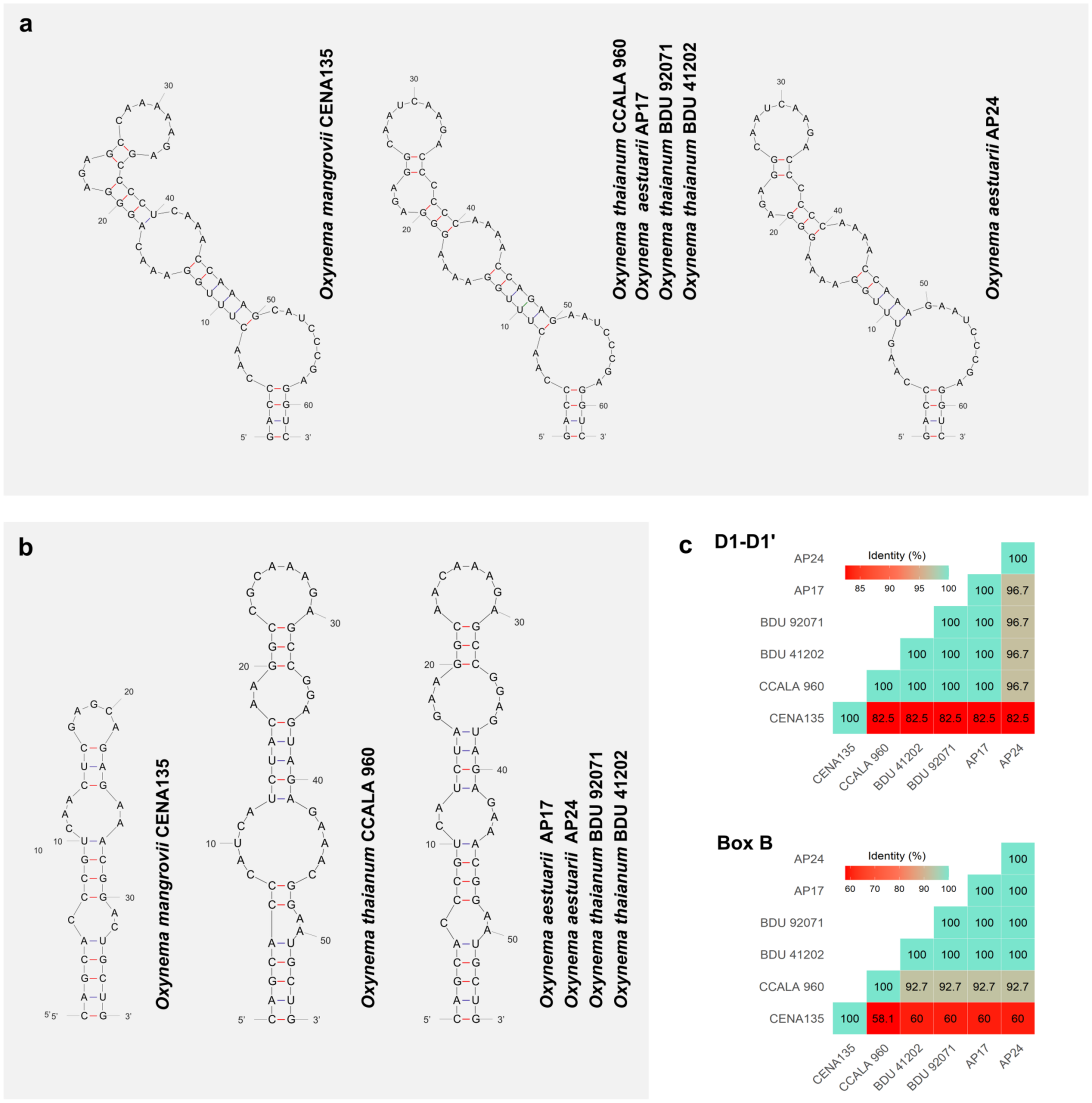
Secondary structures of **(a)** D1-D1’ and **(b)** BoxB helices of the 16S-23S ITS from *Oxynema* strains and **(c)** Identities of its primary structure.

The ITS sequence sizes of six *Oxynema* strains (CENA135, CCALA960, AP17, AP24, BDU 41202, and BDU 92071) ranged from 447 to 547 bp. We identified 14 regions, including genes for tRNA^Ile^ and tRNA^Ala^, according to Iteman et al. (2000) and Table 3. The alignment of the ITS showed differences mainly in D1-D1′, Pre-BoxB, and BoxB regions. The D1-D1’, Box-B, and V3 helices were conserved between all the strains. However, differences were observed in the secondary structures of CENA135 when compared to strains CCALA 960, AP17, and AP24 described by Chakraborty et al. (2018). The secondary structure of D1-D1’ consisted of three bilateral bulges and a terminal loop with a slightly inclined configuration to the right in CENA135 strain (Figure 4a). Box-B revealed a clear separation of CENA135 from *O. thaianum* (CCALA 960, BDU 41202, and BDU 92071) and *O. aestuarii* (AP17 and AP24). The deletion of 18 contiguous nucleotides and six single-nucleotide polymorphisms (SNPs) caused the loss of a bilateral bulge in the Box-B of the CENA135 strain (Figure 4b). The sequence identities among CENA135 and the other *Oxynema* strains showed 82.5% for D1-D1′ and ranged from 58.1 to 60% for Box-B (Figure 4c). Finally, the V3 helix exhibited only one configuration in contrast with that reported by Chakraborty et al. (2018). The 16S–23S ITS sequence has been used for species delineation in cyanobacteria, with D1-D1′, Box-B, and V3 helices being the most used structures to support phylogenetic analyses (Boyer et al., 2001; Johansen et al., 2011). Two of these regions (D1-D1′ and Box-B) were informative for members of the *Oxynema* genus, whose differences were consistent with the 16S rRNA gene evaluations.

**Table 3:** Nucleotide length of the 16S–23S ITS regions of *Oxynema* species.

Strain	Leader	D1-D1′helix	D2 + spacer	D3 + spacer	tRNA ^Ile^ gene	V2	tRNA^Ala^ gene	Pre Box B + spacer	Box B	Post Box B spacer	Box A	D4	V3	D5 + spacer
*Oxynema mangrovii* CENA135	6	**62**	47	16	73	11	72	**33**	**37**	17	12	6	17	**31**
*Oxynema thaianum* CCALA 960	6	**61**	47	16	73	11	72	**46**	**55**	17	12	6	17	**24**
*Oxynema thaianum* BDU 41202	6	**62**	47	16	73	11	72	**48**	**55**	17	12	6	17	**31**
*Oxynema thaianum* BDU 92071	6	**62**	47	16	73	11	72	**48**	**55**	17	12	6	17	**30**
*Oxynema aestuarii* AP17	6	**62**	47	16	73	11	72	**48**	**55**	17	12	6	17	**31**
*Oxynema aestuarii* AP24	6	**62**	47	16	73	11	72	**48**	**55**	17	12	6	17	**31**

ITS regions with differences in length are in bold.

Analyses of morphology, ecology, phylogenomics, 16S rRNA gene, and ITS 16S–23S provided by this study strongly support that the CENA135 strain represents a well-defined species of the *Oxynema* genus, separated from the reference strain *O. thaianum* CCALA 960 and the *O. aestuarii* strains AP17 and AP24. Thus, the results from this study support the proposal of the novel species *Oxynema mangrovii* sp. nov., with *O. mangrovii* CENA135 as the reference strain. This proposal is given under the provisions of the International Code of Nomenclature for algae, fungi, and plants.

### 3.4 Ecological traits

Salinity is an important ecological requirement for *Oxynema* species, influencing their growth and occurrence (Chatchawan et al., 2012; Chakraborty et al., 2018). The strains described so far have been reported from hypersaline habitats or environments with a certain level of salinity, such as mangroves and estuaries. Ecologically, the strains closest to CENA135 were *O. aestuarii* AP17 and AP24, collected from superficial soil biofilms in the Sundarbans mangrove (India). However, the site of occurrence of the Indian strains had a higher salinity level (17.5 ‰, Chakraborty et al., 2018) than the CENA135 isolation site (6.13‰, Rigonato et al., 2013). Meanwhile, *O. thaianum* CCALA960 was recovered from wet salty soil surface or from shallow waters in saltworks of Petchaburi in Thailand (Chatchawan et al., 2012). Likewise, *O. thaianum* BDU 120161 was collected from the muddy soil of the Arambol salt pans from the coastal areas of India (Bhuvaneshwari et al., 2016).

Considering the limited availability of *Oxynema* genomes, genes related to ecological trait resistance were searched on AP17 and CENA135. Both strains contain the *dnaK* operon, responsible for the expression of heat-shock chaperones *dnaK* and *dnaJ*, along with *grpE*, a nucleotide exchange factor associated with *dnaK*’s activity (Gelinas et al., 2003). Considering that mangroves in tropical locations such as Brazil and India experience daily and seasonal temperature fluctuations, *dnaK* can aid in protein refolding, preventing aggregation under heat stress, for continuous metabolic activity (Rajaram et al., 2014). Additionally, high salt concentrations and osmotic pressure can destabilize proteins and cause oxidative damage (Yadav et al., 2022). The *dnaK* operon is important for correct protein conformation, enhancing halotolerance. The sequence identity of the three genes forming this operon was very high between CENA135 and AP17 (98.7 to 100%), and was considerably high (82%) with a *dnaK* copy from *Laspinema olomoucense* D3b.

AntiSMASH annotation revealed the presence of high-identity genes associated with a biosynthetic cluster of a schizokinen siderophore. These small, high-affinity iron-chelating compounds are essential for Fe uptake from insoluble forms (Lynch et al., 2001), which are common in many environmental settings. The cluster showed sequence and structural similarities (Supplementary Figure S2) when compared to the genes found in the cyanobacterium *Nostoc* sp. PCC 7120 (Rudolf et al., 2016) and the bacterium *Sinorhizobium meliloti* 1,021 (Lynch et al., 2001), both known as siderophore producers. Phytoene synthase, an enzyme associated with carotenoid and terpene metabolism, was also found in the antiSMASH analysis, but with low similarity confidence. The biosynthesis of terpene-related compounds in mangrove strains is conceivable, especially since cyanobacteria from tropical soda lakes have been shown to possess highly diverse terpenomes (Machado et al., 2025).

As for metalloproteases and metal transporters, including cobalt (*cbiM* and *cbiQ*), magnesium and Co (*corA*), zinc (*FtsH*), and potassium (*TrkA*), multiple copies with very high identity (>96%) were found between both *Oxynema* genomes. These proteins are essential for microorganisms inhabiting mangrove areas, which are rich in decaying organic matter and end up accumulating metals (Marchand et al., 2011). These metalloproteases can function in synergy with chaperones like *dnaK,* degrading damaged proteins potentially accumulated due to metallic stress (Sanders et al., 2023). As for metal transporters, they are required both for metal acquisition (functioning as essential metabolic cofactors) and metal detoxification (Baptista and Vasconcelos, 2006). Since cobalt tends to be less bioavailable in estuarine and mangrove sediments (Fernandes et al., 2014), specialized uptake systems can be extremely advantageous. Also, Mg and K are vital for intracellular osmotic balance (Bremer and Krämer, 2019; Wendel et al., 2022), hence highlighting the importance of possessing these genes when inhabiting a challenging environment such as mangroves.

Finally, cation:anion antiporters, such as Na^+^/H^+^ or K^+^/H^+^,were also found in *Oxynema* genomes. CENA135 had more copies of antiporters (15) than AP17 (10), exemplifying the plasticity between species of the same genera. *NhaP* is an important antiporter for pH homeostasis and salt tolerance, particularly in a tidal saltwater influx condition such as the one experienced in a mangrove (Resch et al., 2011). The copies found in both *Oxynema* were similar to *NhaP* from *Laspinema palackyanum* D2c (82.64% sequence identity), a strain isolated from a mat covering an ephemeral shallow rainfall puddle on soil (Dvořák et al., 2024).

## 4 Description of *Oxynema mangrovii* sp. nov. Apaza-Castillo et al.

Entangled trichomes grow by forming blue-green mats with a deep blue color in low-light conditions. Trichomes are straight or slightly flexuous, attenuated to the ends, and slightly constricted at the cross-walls. A sheath is not observed in culture conditions. Vegetative cells are shorter than wide, occasionally isodiametric, 1.9–5.1 μm long (3.3 μm on average), 2.7–3.4 μm wide (3.1 μm on average), and with a cell length/width ratio of 0.6 to 1.1. The cell content is blue-green, granulated, and with a central vacuole sometimes present. Mature apical cells are elongated, narrowed, sharply pointed, usually bent, and 3.2–8.7 μm long. Necridial cells are observed before the trichomes break into hormogonies. The trichomes are intensely motile by gliding.

- Epithet etymology: *mangrovii* (man.gro’vii. N. L. gen. n.), Latin noun corresponding to “of the mangrove” (the genitive of *mangrovium*), referring to the environment inhabited by the strain collected.
- Type locality: Cardoso Island, municipality of Cananéia, São Paulo State (25°05′02”S, 47°57′42”W).
- Habitat: Mangrove wet soil.
- Holotype: Exsiccate accession number SP428477, deposited at “Maria Eneyda P. Kauffman Fidalgo” Herbarium of the Institute of Botany, São Paulo state, Brazil.
- Reference strain: *Oxynema mangrovii* CENA135.
- Genomic sequence available: NCBI/GenBank accession number JAEOXH000000000, GCA_016632315.1.

## 5 Conclusion

This study provides a detailed description of *Oxynema mangrovii* sp. nov. Apaza-Castillo et al. The genome assembly of this new species is presented, highlighting the presence of specific environmental resistance-related genes. The taxonomic placement of *O. mangrovii* CENA135 is provided through a polyphasic approach including morphological description, 16S rRNA gene sequence identity and phylogeny, 16S–23S ITS secondary structures, phylogenomics, digital DNA–DNA hybridization (dDDH), and whole-genome average nucleotide identity (ANI). The sum of all this data placed the newly described species *O. mangrovii* CENA135 as part of the Oscilatoriales order, with a common ancestry to all other *Oxynema* strains and within a clade that is taxonomically close to *Laspinema* and *Oscillatoria.* As only the second assembled genome of this genus, this study expands knowledge concerning cyanobacterial diversity from underexplored habitats, such as mangroves.

## Data Availability

The datasets presented in this study can be found in online repositories. The names of the repository/repositories and accession number(s) can be found in the article/Supplementary material. The genetic data presented in the study are deposited in the NCBI repository, accession numbers HQ730084 and GCA_016632315.1.

## Footnotes

1
https://ggdc.dsmz.de/ggdc.php

2
http://eddylab.org/software/ssu-align/
