# Supplementary material for: Oxynema mangrovii sp. nov., a new filamentous species (Oscillatoriales, Cyanobacteria) from Atlantic forest mangrove
Source: Front Microbiol. 2026 Jan 20;16:1709185. doi: 10.3389/fmicb.2025.1709185 (PMC12895190; doi:10.3389/fmicb.2025.1709185)
Supplement: Supplementary file 1 [file Supplementary_file_1.docx]

Supplementary Material

***Oxynema* *mangrovii* sp. nov., a new filamentous species (Oscillatoriales, Cyanobacteria) from Atlantic Forest mangrove**

**Gladys A. Apaza-Castillo^1,2^, Rafael B. Dextro^1^, Ana P. D. Andreote^1^ , Bruno C. E. Souza^1^, Guilherme K. Hosaka^2^ , Endrews Delbaje^1^, Luis H. Z. Branco^3^, Diego M. Riaño-Pachón^1^, Marli F. Fiore^1*^**

^1^University of São Paulo (USP), Center for Nuclear Energy in Agriculture (CENA), Avenida Centenário 303, 13400-970 Piracicaba, SP, Brazil

^2^ University of São Paulo (USP), Luiz de Queiroz College of Agriculture, Piracicaba, São Paulo, Brazil

^3^ São Paulo State University, Institute of Bioscience, Languages and Exact Sciences, 15054-000 São José do Rio Preto, SP, Brazil

**Correspondence**:

Marli F. Fiore

[fiore@cena.usp.br](mailto:fiore@cena.usp.br)

# Supplementary Figures

# Figure S1. Map with geographical coordinates of the sampling location in Ilha do Cardoso, located at the coast of São Paulo state, in the Southeast region of Brazil.

# Figure S2. Genetic organization of the putative cluster involved in siderophore biosynthesis and transport in *Oxynema mangrovii* CENA135 (A), along with the genetic clusters of *Nostoc* sp. PCC 7120 (B) and *Sinorhizobium meliloti* 1021 (C), known siderophore producers.

# Figure S3. 33-Kmer distribution of *Oxynema mangrovii* CENA135 Illumina DNA reads.

# Figure S4. Heatmap of the whole-genome average nucleotide identity (ANI) highlighting the relationship between *Oxynema mangrovii* CENA135 (in bold) and seven other strains with taxonomic relevance.


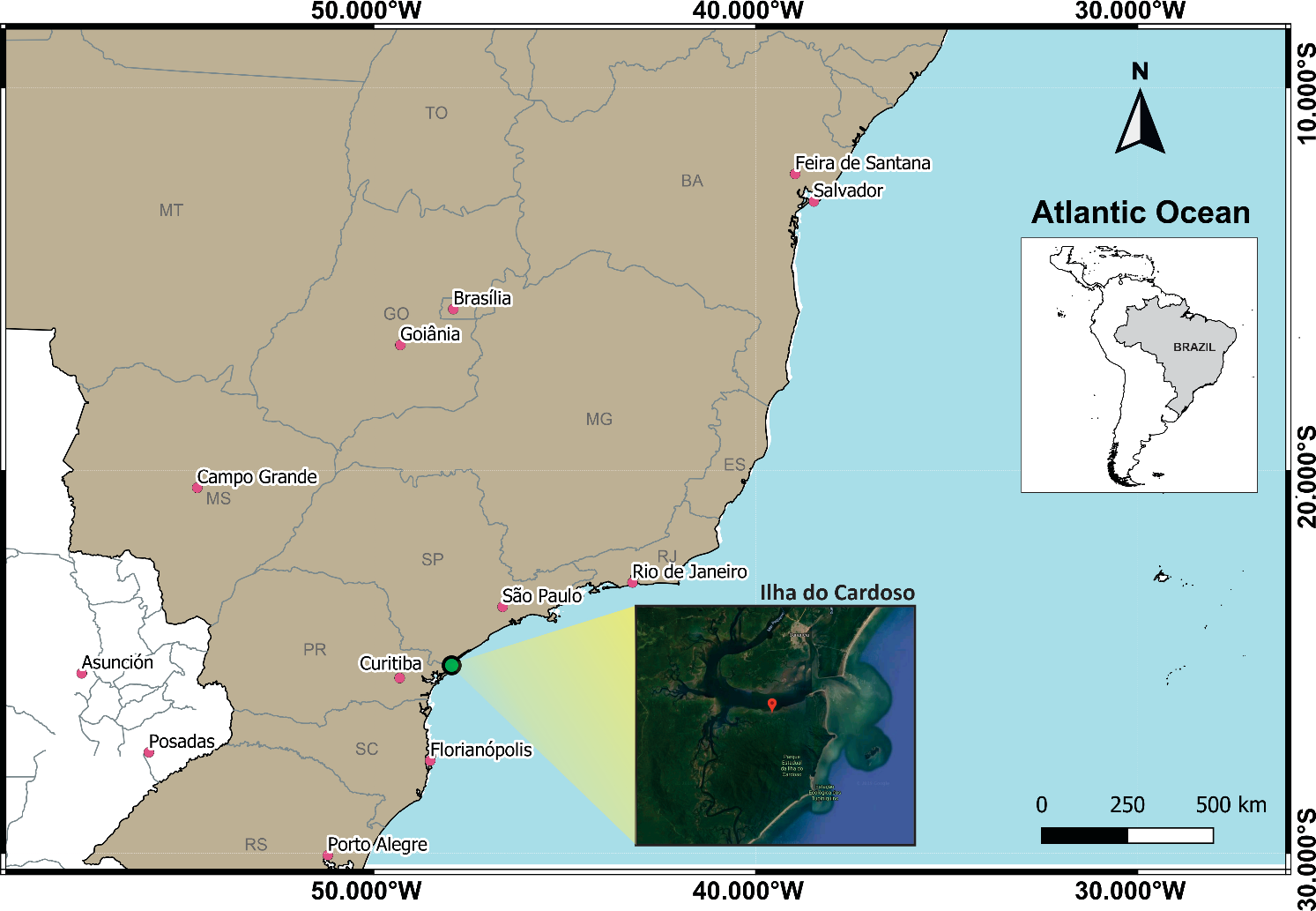


**Supplementary Figure 1.** Map with geographical coordinates of the **s**ampling location in Ilha do Cardoso, located at the coast of São Paulo state, in the Southeast region of Brazil.


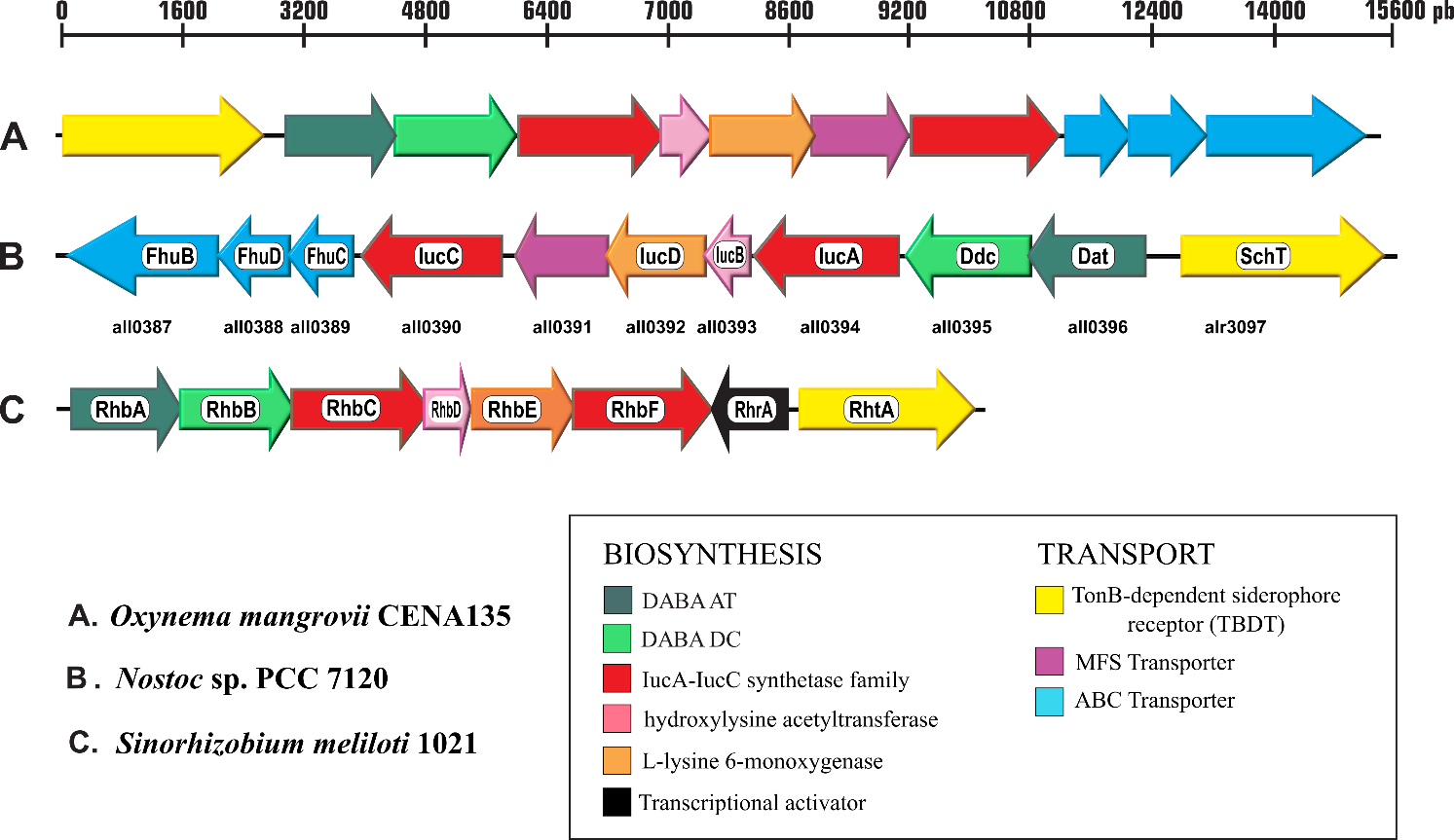


**Supplementary Figure 2.** Genetic organization of the putative cluster involved in siderophore biosynthesis and transport in *Oxynema mangrovii* CENA135 (A), along with the genetic clusters of *Nostoc* sp. PCC 7120 (B) and *Sinorhizobium meliloti* 1021 (C), known siderophore producers.


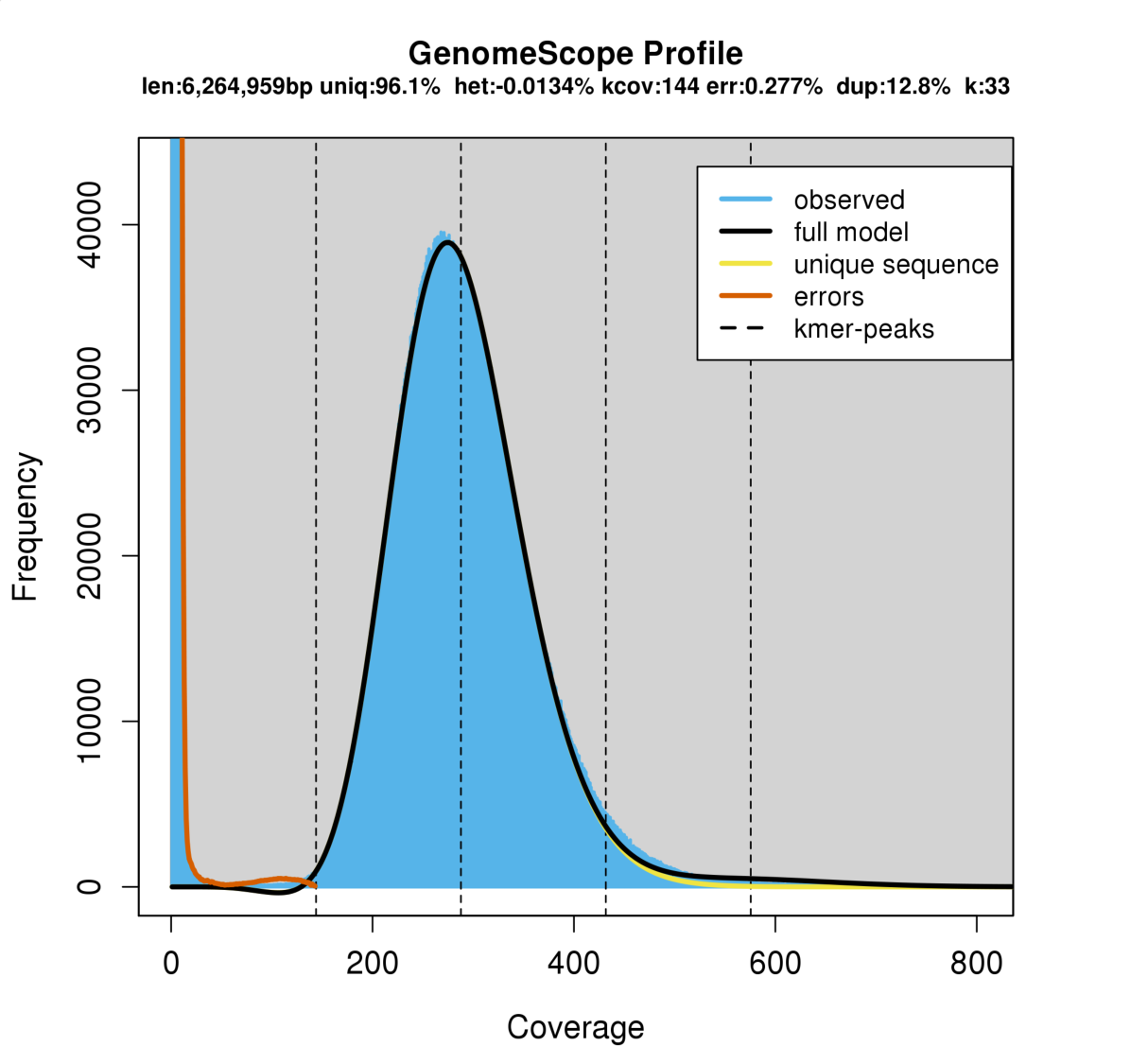


# Supplementary Figure 3. 33-Kmer distribution of *Oxynema mangrovii* CENA135 Illumina DNA reads.


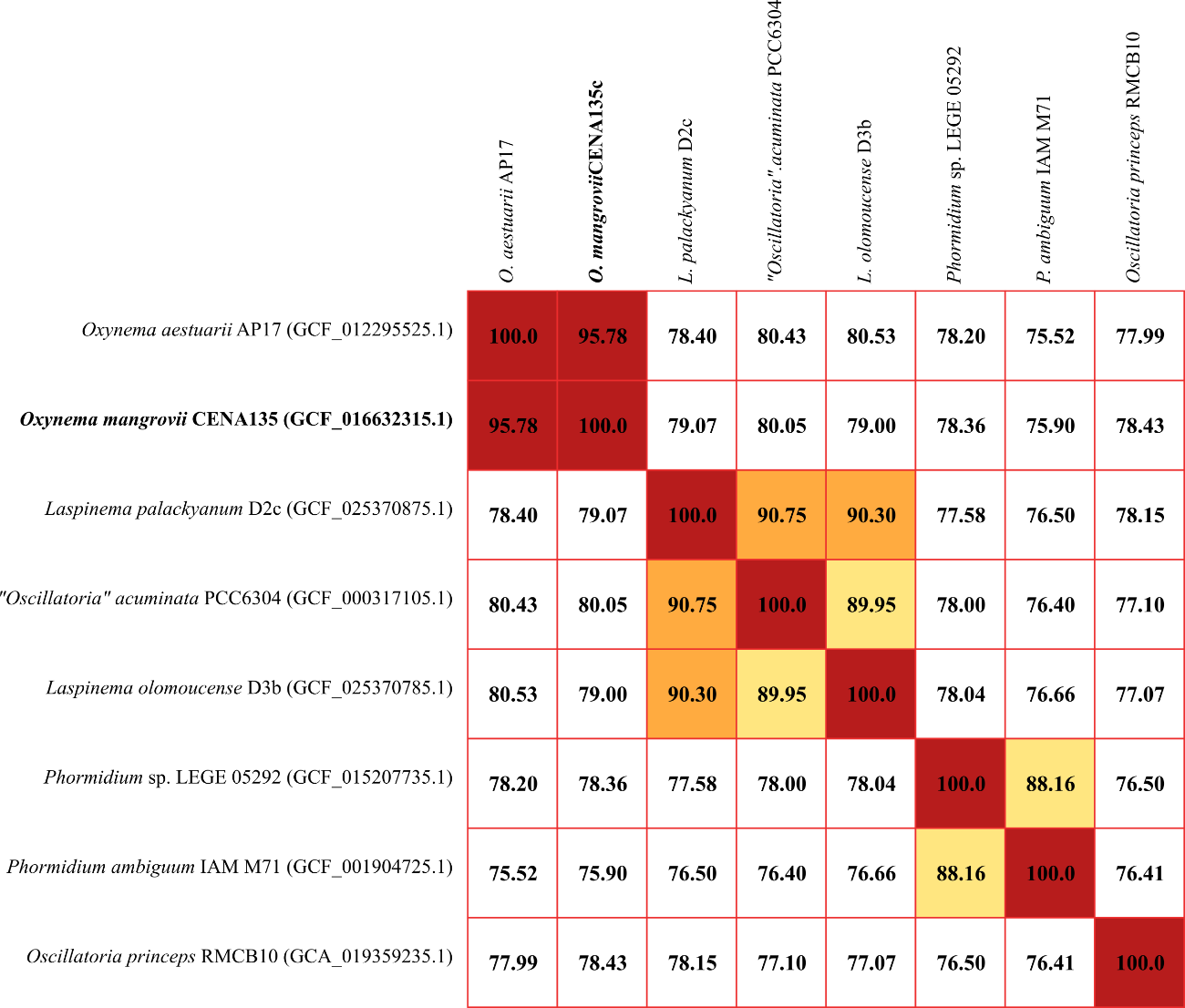


**Supplementary Figure 4.** Heatmap of the whole-genome average nucleotide identity (ANI) highlighting the relationship between *Oxynema mangrovii* CENA135 (in bold) and seven other strains with taxonomic relevance.

# Supplementary Tables

# Table S1. Metrics of individual and consensus assemblies of *Oxynema mangrovii* CENA135 genome.

**Table S2.** Morphometric, ecological and genomic comparisons of *Oxynema aestuarii* AP17 and *Oxynema thaianum* CCALA960 in regards to *Oxynema mangrovii* sp. nov. CENA135.

**Supplementary Table 1.** Metrics of individual and consensus assemblies of *Oxynema mangrovii* CENA135 genome.

| **Metrics** | **1** | **2** | **3** | **Consensus** |
| --- | --- | --- | --- | --- |
| Assembly Software | SPAdes | Platanus | MetaSPAdes  + MetaBAT2 | Metassembler |
| Assembly size (bp) | 6,268,060 | 6,032,431 | 6,260,271 | 6,241,108 |
| Number of scaffolds | 155 | 265 | 10 | 11 |
| N° of scaffolds >1000 pb | 16 | 39 | 10 | 9 |
| Largest scaffold | 3,591,057 | 1,218,266 | 2,735,654 | 3,591,108 |
| N50 | 3,591,057 | 431,201 | 927,442 | 3,591,108 |
| L50 | 1 | 5 | 2 | 1  51.6 |
| GC % | 51.6 | 51.6 | 51.6 |  |
| Completeness (%) | 99.29 | 99.11 | 98.35 | 99.29 |
| Contamination (%) | 1.45 | 1.69 | 3.73 | 1.22 |

**Supplementary Table 2.** Morphometric, ecological and genomic comparisons of *Oxynema aestuarii* AP17 and *Oxynema thaianum* CCALA960 in regards to *Oxynema mangrovii* sp. nov. CENA135.

| Strain | Cell width (µm) | Cell length (µm) | Motility | Sheath | Apical cell shape | Culturing pH | Culturing temperature (°C) | GC (%) | ANI (%) compared to CENA135 | dDDH (%) compared to CENA135 | 16S (%) compared to CENA135 |
| --- | --- | --- | --- | --- | --- | --- | --- | --- | --- | --- | --- |
| *Oxynema mangrovii* sp. nov. CENA135 | 2.7 - 3-4 | 1.9 – 5.1 | Yes | Absent | Elongated, pointed | 6.8 | 23 ± 1 | 51.6 | - | - | - |
| *Oxynema aestuarii* AP17 | 2 – 2.45 | 1.5 – 2.5 | No | Present | Elongated, pointed | 7.5 | 25 ± 2 | 51.5 | 95.7 | 62.8 | 98.4 |
| *Oxynema thaianum* CCALA960 | 7.9 - 8 | 2.6 – 2.7 | No | No Data | Elongated, pointed | 7.9 | 25 | No Data* | No Data* | No Data* | 98.4 |

* *Oxynema thaianum* CCALA960 does not have a sequenced genome available
